# Bridging population genetics and the metacommunity perspective to unravel the biogeographic processes shaping genetic differentiation of *Myriophyllum alterniflorum* DC.

**DOI:** 10.1038/s41598-019-54725-7

**Published:** 2019-12-02

**Authors:** Jorge García-Girón, Pedro García, Margarita Fernández-Aláez, Eloy Bécares, Camino Fernández-Aláez

**Affiliations:** 1 Group for Limnology and Environmental Biotechnology, Area of Ecology, Universidad de León, Campus de Vegazana, León, Spain; 2 Department of Molecular Biology, Universidad de León, Campus de Vegazana, León, Spain

**Keywords:** Freshwater ecology, Molecular ecology

## Abstract

The degree to which dispersal limitation interacts with environmental filtering has intrigued metacommunity ecologists and molecular biogeographers since the beginning of both research disciplines. Since genetic methods are superior to coarse proxies of dispersal, understanding how environmental and geographic factors influence population genetic structure is becoming a fundamental issue for population genetics and also one of the most challenging avenues for metacommunity ecology. In this study of the aquatic macrophyte *Myriophyllum alterniflorum* DC., we explored the spatial genetic variation of eleven populations from the Iberian Plateau by means of microsatellite loci, and examined if the results obtained through genetic methods match modern perspectives of metacommunity theory. To do this, we applied a combination of robust statistical routines including network analysis, causal modelling and multiple matrix regression with randomization. Our findings revealed that macrophyte populations clustered into genetic groups that mirrored their geographic distributions. Importantly, we found a significant correlation between genetic variation and geographic distance at the regional scale. By using effective (genetic) dispersal estimates, our results are broadly in line with recent findings from metacommunity theory and re-emphasize the need to go beyond the historically predominant paradigm of understanding environmental heterogeneity as the main force driving macrophyte diversity patterns.

## Introduction

The degree to which dispersal limitation interacts with environmental filtering has intrigued metacommunity ecologists and population geneticists since the beginning of both research disciplines^1^. Today, metacommunity ecology has rapidly become a dominant framework through which ecologists understand the natural world^2^. Both, population genetics and metacommunity ecology, posit that it is not only the local environment that dictates patterns of species distributions, but these patterns also depend on processes such as the movement of organisms at the regional scale^3,4^. Each has generated an impressive body of theoretical and empirical research over the past two decades, yet dispersal processes operating in aquatic organisms remain little explored^5^. This deficit is a major hindrance to our understanding of dispersal as a force structuring regional patterns of biodiversity^6^, and is also the main reason why ecologists usually need to rely on proxies for dispersal. Recent simulation studies have shown that this typical coarse interpretation of spatial-based processes, which is primarily derived from the use of orthogonal spatial eigenvectors (MEM^7^) in variation partitioning analysis, is to some degree flawed, resulting in greatly inflated estimates of the role of environmental filtering^8^. Consequently, much of modern freshwater ecology is founded on the principle of environmental determinism and its findings are still subject to revision. In this vein, the recent macroecological study of Alahuhta *et al*.^9^ (using partial redundancy analysis and the standard variation partitioning approach) showed that environmental filtering overrode the effects of potential connectivity in explaining local communities across the world. However, such a statistical approach is highly correlative and was recently shown to overlook the role of dispersal-related processes on species distributions at a landscape level^8^.

Recent recognition of the limitations affecting the traditional community-based approach to assess the relationships between dispersal limitation and environmental filtering called for the development of more sophisticated empirical methods^10^. In this context, it is important that ecologists take advantage of modern techniques that have the potential to inform robust mechanistic models^11^. These techniques include molecular tools, which have been historically employed to study gene flow and potential dispersal limitation in biological populations^12,13^. Since gene flow estimations are certainly superior to coarse proxies of dispersal, understanding how environmental and geographic factors influence the genetic structure of biodiversity is becoming one of the most fundamental issues for population genetics^14^ and also one of the most challenging avenues for metacommunity ecology^10^. Consequently, progress in the synergistic potential of population genetics and metacommunity ecology may help in elucidating the degree to which dispersal limitation interferes with the local environment in determining geographic patterns of biological diversity.

Landscape genetic scenarios, including ‘isolation by distance’ (IBD) and ‘isolation by the environment’ (IBE), rely on inferring the role of dispersal limitation and environmental variation from observed patterns of genetic structure. The theory of isolation by distance describes the local accumulation of genetic differences when dispersal among populations is limited by geographic factors, and therefore gene flow is inversely proportional to the distance between populations^4,15^. Thus, genetic differentiation is the result of drift acting within populations more quickly than it is mitigated by gene flow among populations^14,15^.

Conversely, when genetic differentiation positively aligns with environmental dissimilarity among sites, a pattern of isolation by environment emerges^4^. This model suggests that environmental variables can influence the colonisation success of individuals and groups of individuals via environmental filtering, with higher effective gene movements among similar environments^4,14^. Hence, regional variation in the environment may influence species-specific colonisation rates and establishment success when geographic distance allows dispersing immigrants to reach nearby habitat patches^14^. A classic example of this scenario in the freshwater realm comes from plants growing on and near the reaches of lakes and rivers, where local adaptations to different sediment and soil types have occurred^4^. Both patterns of IBD and IBE are usually present simultaneously in nature^4^ and represent one of the most important approaches with which to assess the relative importance of geographic distance and environmental heterogeneity in shaping patterns of dispersal and genetic variation^14,16,17^.

Relatively few empirical studies have examined the contribution of dispersal limitation and environmental filtering on macrophyte genetic divergence^16–18^. The few existing studies^16–19^ seem to reveal simultaneous IBD and IBE patterns in shaping the genetic structure of different aquatic macrophyte species. Importantly, the interaction between spatial and environmental dynamics in structuring macrophyte genetic differentiation is broadly in line with recent findings from metacommunity theory^20,21^, and emphasizes the need to go beyond the historically predominant paradigm of understanding environmental specificity as the main force driving macrophyte gene flow patterns^22,23^.

Here, we present an analysis of geographic genetic variation using microsatellite markers on a total of 11 populations of *Myriophyllum alterniflorum* DC. (2n = 14) from 11 ponds located in the Iberian Plateau. More specifically, our main aims were to: **(i)** explore geographic patterns of population genetic structure and gene flow in the alternateflower watermilfoil; **(ii)** assess the influence of geographic distance and environmental dissimilarity on genetic differentiation of *M. alterniflorum* in Mediterranean pond environments; and **(iii)** disentangle if results obtained through population genetic methods match modern perspectives of metacommunity theory in these landscapes. Based on evidence from previous genetic studies on aquatic macrophytes^16–19^, we expected both geographic distance and environmental filtering to influence genetic variation of *M. alterniflorum* at the regional scale (**H1**). We also assumed that habitat fragmentation in Mediterranean landscapes would require that gene flow occurs primarily between neighbouring populations (**H2**), supporting recent metacommunity empirical research^20,21^ (**H3**) that suggests that spatial structuring accounts for much of the variation in aquatic macrophyte diversity patterns.

Since no academic work has yet examined the actual patterns of gene flow in aquatic macrophytes from a Mediterranean perspective, we hope that the baseline genetic information of our work may provide ground-breaking insights into the role of geographic isolation and environmental filtering on macrophyte metapopulation structuring. Similarly, our findings may have important and widespread implications for integrating population genetics into the full inference space of metacommunity ecology, helping us obtain a deeper understanding of whether or not spatial processes may hinder aquatic macrophytes from tracking environmental variation at the regional scale. It is important to emphasise that our study is limited to a single macrophyte species, and since most metacommunities comprise dozens to hundreds of species, findings should therefore be handled with caution. This is because, although the fundamental units are analogous (taxa in communities, alleles in populations), population genetics and metacommunity ecology use different routines and approaches to disentangle geographic patterns of biological diversity^3,4^. However, it is now becoming feasible to compare community assessments with genetic variation of single or a few species^10^. Whatever the case, we strongly believe that the alternateflower watermilfoil offers an ideal example for us to assess the spatial genetic patterns of aquatic macrophytes because it occurs irrespectively in still or slow-moving water of lakes, ponds and rivers and it is also widely distributed in different biogeographic realms of the Earth^19^.

## Results

### Genetic diversity

**Figure 1:**
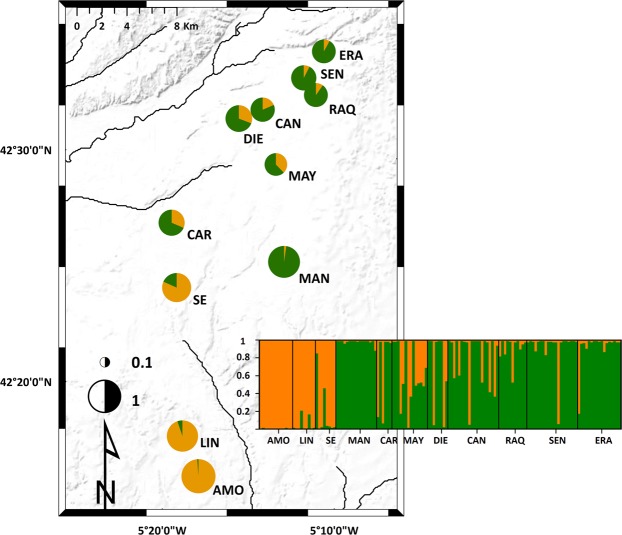
Estimated genetic structure of *Myriophyllum alterniflorum* populations inferred by a Markov chain Monte Carlo clustering (STRUCTURE) at the individual level (*K* = 2). Black lines indicate different population origins. Pie charts represent the probability of assignment to one of the two clusters (orange: southern cluster; green: northern cluster). The areas of the pie charts are proportional to the mean F_ST_ values over loci. The colour scales are used in Fig. 2.

A total of 142 genets were revealed in the samples. All of the obtained genotypes were population specific. Thus, no clones were found between or within populations, suggesting that dispersal of vegetative propagules is uncommon in *M. alterniflorum*. Total genetic diversity (H_T_) and mean within-population diversity (H_S_) were 0.65 and 0.56, respectively. Considering all study populations, the number of genets, the mean number of alleles (N_a_) and the mean effective number of alleles (N_e_) ranged from 6.0 to 20, 2.9 to 5.4, and 2.0 to 3.0, with a mean of 13, 4.4 and 2.6, respectively. The mean observed heterozygosity (H_o_) and the mean unbiased expected heterozygosity (uH_E_) were 0.60 and 0.56, varying between 0.47–0.71 and 0.45–0.66, respectively (Table 1). The pairwise Nei’s unbiased genetic distance (D_A_; Supplementary Table S1) ranged from 0.01 to 0.62, with comparisons involving individuals from AMO-MAN and RAQ-ERA-CAN presenting the greatest and the smallest genetic dissimilarity, respectively (see Fig. 1 for abbreviations). The population-specific F_IS_ varied from −0.25 to 0.15, with an average of −0.02 (Table 1). The mean values of F_IT_ and F_ST_ were 0.11 and 0.13, respectively, suggesting moderate genetic differentiation and limited inbreeding between populations.

**Table 1:** Results of genetic diversity measures for natural populations of *Myriophyllum alterniflorum*, geographic origins (UTM) and values of the first two principal components (PCA1, PCA2) to the environmental features in the study ponds.

Populations	Latitude	Longitude	Population size	Number of genotypes	N_a_	N_e_	H_o_	uH_E_	F_IS_	PCA1	PCA2
AMO	4682078	310393	13	13	4.2	2.8	0.58	0.62	0.13	501	405
LIN	4685289	309108	9	9	4.1	2.8	0.57	0.52	0.15	−281	142
SE	4697150	308647	8	8	4.3	3.0	0.68	0.66	−0.01	111	−217
MAN	4699206	317237	16	16	4.0	2.0	0.49	0.45	−0.07	−144	−29
CAR	4702335	308243	6	6	2.9	2.2	0.46	0.55	0.10	362	−154
MAY	4706991	316586	14	14	5.0	3.0	0.63	0.65	0.01	161	−35
DIE	4710657	313622	8	8	3.8	2.2	0.47	0.52	0.14	162	−274
CAN	4711368	315523	20	20	4.6	2.3	0.65	0.53	−0.25	−212	24
RAQ	4712540	319786	11	11	5.1	2.7	0.68	0.63	−0.10	−202	70
SEN	4713904	318812	20	20	5.4	3.0	0.71	0.63	−0.15	−197	−4
ERA	4716025	320422	17	17	5.0	2.7	0.71	0.63	−0.13	−262	72
Average			13	13	4.4	2.6	0.60	0.59	−0.02		
Standard deviation			4	4	0.2	0.1	0.03	0.02	0.05		

Number of ramets (population size), number of genets (number of genotypes), mean number of alleles (N_a_), mean number of effective alleles (N_e_), observed heterozygosity (H_o_), unbiased expected heterozygosity (uH_E_) and inbreeding coefficient (F_IS_).

### Population genetic structure

**Figure 2:**
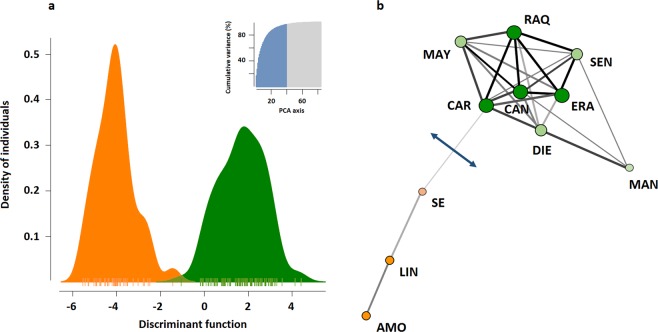
(**a**) Discriminant analysis of principal components (DAPC) showing the individual density plot on the first discriminant function (*k* = 2). The top right histogram illustrates the amount of variation explained by the principal components (PCAs = 40). (**b**) Simplified network identified by EDENetworks between nodes (sampling sites). Line thickness is proportional to linkage strength and node size is proportional to the number of linkages for each population. The blue line indicates the position of the single barrier to gene flow for more than half the loci set identified by the Monmonier’s algorithm after 1,000 bootstrap replicates.

The STRUCTURE analysis (Fig. 1) suggested K = 2 as the optimal number of clusters based on the second order rate of change of the likelihood function, *∆K* (Supplementary Fig. S1). One geographical group consisted of populations from the northern part of the study area (ERA, SEN, RAQ, CAN, DIE, MAY, CAR and MAN), while the other cluster comprised populations from the south (SE, LIN and AMO). Four ponds (MAN, SE, LIN and AMO) showed the greatest genetic divergence (F_ST_; Fig. 1). Discriminant Analysis of Principal Components (DAPC) was performed on the first 40 principal components to capture nearly 90% of the total genetic variation. Results of the DAPC analysis (Supplementary Table S2) were analogous to those from the STRUCTURE. In summary, the individual density plot on the first discriminant function revealed a clear separation between the two population clusters identified by the Bayesian clustering approach (Fig. 2a).

The minimum spanning network (MSN) plotted by EDENetworks (Fig. 2b) revealed several distinct topological features. The network drawn from the distribution of alleles between populations indicated a clear separation between the two clusters identified by the STRUCTURE and DAPC analyses. Similarly, a consistent patterns for barriers to gene flow was observed with the Monmonier’s algorithm (Fig. 2b). The single geographic boundary was the one separating the three southern ponds (LIN, AMO and SE) from all populations north of SE. Consequently, MSN and Monmonier’s algorithm seemed to confirm our previous results, suggesting that the three southern populations (SE, LIN, AMO) exhibited a strong isolation by distance and experienced very limited gene flow from northern ponds. Conversely, populations from the northern cluster were likely to be relatively well connected by a number of links, with CAN functioning as a connectivity provider for nearby ponds.

### Landscape genetic analysis

After the PCA-based model selection procedure, we kept the first two axes as synthetic environmental variables since these two principal components explained ~98% of the variance in the environmental attributes. The first axis was closely associated with turbidity, nutrient content and hydroperiod length, while pH and conductivity had the largest independent contribution for variation in the second axis (Supplementary Table S3).

**Figure 3:**
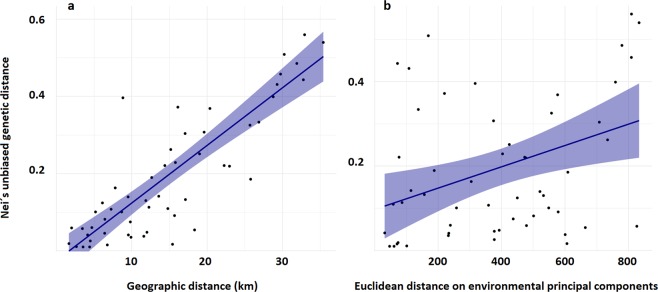
Scatter plots of Mantel tests showing the relationships between genetic differentiation, geographic distance (**a**) and environmental dissimilarity (**b**).

Our analyses by Mantel test suggested a significantly positive correlation between genetic and geographic distances (*r* = 0.82, *p* = 0.001), whereas non-significant associations were detected between genetic differentiation and environmental dissimilarity (*r* = 0.50, *p* = 0.1). When the influence of the environmental factor was controlled, the genetic-spatial association remained highly significant (*r* = 0.78, *p* = 0.001; Table 2; Fig. 3). According to the MMRR, geographic distance had the highest regression coefficient (*β* = 0.71, *p* = 0.005), while the effects of environmental heterogeneity were again statistically non-significant (*β* = 0.13, p = 0.86).

**Table 2:** Simple and partial Mantel tests showing correlations between genetic distance, geographic distance and environmental dissimilarity.

Landscape feature	Controlled	r	*p*
Geographic distance		**0.82**	**0.001**
Environmental dissimilarity		0.50	0.06
Geographic distance	Environmental dissimilarity	**0.78**	**0.001**
Environmental dissimilarity	Geographic distance	0.31	0.11

Significant values are presented in bold.

## Discussion

Compared with previous works on patterns of genetic differentiation in aquatic macrophytes^23–26^, the present study was performed along a relatively wide environmental gradient in a Mediterranean landscape. Estimates of genetic diversity (i.e. total diversity, H_T_; mean within-population diversity, H_S_; observed heterozygosity, H_o_; and unbiased expected heterozygosity, uH_E_) for *M. alterniflorum* populations were somewhat high compared to the average values reported for other aquatic macrophytes with similar life history traits at both the species and the population level^17,18,27^. The possible reason for the relatively high genetic variation uncovered here may be explained in terms of high landscape heterogeneity and the wide range of local ecological conditions of the study ponds^28^. Likewise, all the obtained genotypes were population-specific, suggesting a high rate of recombination due to sexual reproduction and uncommon interpopulation dispersal of vegetative propagules^29^. In this regard, García-Girón *et al*.^30^ recently showed that seeds of *M. alterniflorum* were far more frequently encountered in surface sediment than its vegetative plant fragments, supporting our assumption that the spread of vegetative propagules is a relatively rare event. In any case, these patterns of genetic variability suggest that Mediterranean ponds are important reservoirs of genetic diversity^25^.

In spite of the relatively short distances between ponds (mean pairwise geographic distance ~ 15 km), and following our expectations (**H2**), populations clustered into genetic groups that mirrored their geographic patterns. Indeed, the Bayesian clustering approach and the discriminant analysis of principal components found only two genetic clusters in the data, one comprised of the eight populations north of SE and the other comprised of SE and the two populations south of this pond. This grouping was supported by the EDENetwork analysis, which also found modest levels of population connectivity. Sites spanning the northern cluster were relatively well connected, with the centrally located CAN being an important node linking several sites (Fig. 2b). This finding highlights the degree to which stepping-stone ponds may function as habitat connectivity providers for short-distance seed exchanges among otherwise isolated habitat patches^4^. By contrast, sites south of SE showed less population connectivity and seemingly were not linked to the northernmost populations, suggesting that gene flow was not sufficient to keep a single, panmictic, spatially extended population throughout the study range. This finding is intuitive given that ponds spanning the southern cluster were largely isolated from the northernmost sites (see Fig. 1), so the further apart ponds were, the less likely they were to share a similar allelic composition. This is especially true when little or no physical connection via flowing water exists between the sites^20^ (see below). Accordingly, the Monmonier’s algorithm indicated that there was a single barrier to gene flow, isolating the southernmost populations front the rest of the species’ range. This means that geographic limits will decrease the probability of effective dispersal among distant populations and thus enhance differentiation through genetic drift^31^.

It is generally accepted that IBD and IBE are the main scenarios structuring genetic divergence in natural populations^4^. However, only a few empirical studies have assessed the relative role of geographic distances and environmental factors in shaping genetic patterns of aquatic macrophyte populations^32^, and most of them come from temperate Europe^33,34^ and Asia^16–18^. To the best of our knowledge, no academic work has yet examined the actual patterns of gene flow in aquatic macrophytes from Mediterranean landscapes and whether there is a prevailing scenario with respect to spatial and environmental gradients. This particular situation has led Mediterranean ecologists to understand organism-landscape interactions in terms of the well-established theory of metacommunity organization, in which the interplay of spatial processes and local environmental forces is determined by performing robust, mechanistic models^20,21^. However, such an indirect approach produces information on the influence of spatial processes, environmental filtering, and both in combination, from coarse proxies of dispersal, such as eigenfunction spatial analyses^7^ and distance decay functions^35^, potentially overfitting the environmental component^8^. Hence, incorporating more realism in the genetic architecture of dispersal may play an important role in advancing our understanding on how landscape variation shapes the distribution of aquatic macrophytes in this kind of highly fragmented environments. Since extensive Mediterranean environments are found in several locations worldwide and are relatively sensitive to climate and land-use change^36^, addressing this knowledge gap has important, widespread implications.

In our study, a strong association between genetic and geographic distances revealed a pattern of IBD across the distributional range of *M. alterniflorum* in northern Spain, partially confirming our main hypothesis (**H1**). Under this scenario, increasing geographic distances among populations is expected to lead to enhanced genetic differentiation^4–15^, which essentially requires that gene flow occurs primarily between neighbouring populations^31^. Several previous studies revealed strong interpopulation genetic divergence and patterns of IBD in aquatic macrophytes at the continental scale^16–18^. However, to our knowledge, our study is the first empirical evidence that links macrophyte genetic variation and geographic isolation at the regional scale in Mediterranean landscapes. Globally, our study suggests that the dispersal of *M. alterniflorum* individuals among Mediterranean ponds was limited by geographic isolation and argues in favour of the classical stepping-stone model in which gene flow is mostly restricted to neighbour populations^31^.

Wind, water and animals are the three main agents of dispersion for aquatic macrophytes, but their relative roles are very different. For example, Sommers *et al*.^5^ found that hydrochory is a common means of long-distance dispersal in wetland species, facilitating gene exchange among geographically isolated populations and reducing the effect of founder events and genetic drift^37^. In fact, water dispersal has long been recognized as the main reason for the wide distribution of freshwater macrophytes^38^. Given the few hydrological connections among the study ponds^39^, wind and animals were likely to play the primary role for inter-population gene flow, transporting pollen, seeds and vegetative propagules to other ponds over the landscape^20^. Since wind-mediated gene flow seems to be effective only for distances of less than 1 km^40^, anemochorous dispersal may have failed to homogenize allele frequencies across distant populations. This reasoning is also in agreement with other experimental studies that have investigated spatial genetic structure of submerged macrophyte species in wetlands^34^. On the other hand, some waterbirds are known to play an important role in the seed dispersal of watermilfoils^41^. However, given the small populations of indigenous and migratory waterfowl in the study area^39^, we speculate that bird-mediated dispersal was likely to be of minor importance for gene flow patterns. Therefore, the single means of dispersal and lack of mediators (i.e. water and birds) may have enhanced genetic differentiation of *M. alterniflorum* populations in Mediterranean ponds, which could explain the greater prediction of IBD on genetic divergence as a result of genetic drift and dispersal limitation^16^.

Since the traditional view of macrophyte community studies is founded on the principle of environmental determinism^9,42^, the results we report here may seem surprising. However, recent empirical research^8,20^ suggests that spatial structuring and environmental control together accounts for much of the variation in aquatic macrophyte communities at different spatial scales and geographic areas. For example, using a novel combination of metacommunity assembly modelling and multivariate multiscale codependence analysis, García-Girón *et al*.^20^ showed that dispersal limitation acted in concert with species sorting to influence macrophyte community assembly processes in Mediterranean landscapes. In this regard, a growing number of studies^20,21,43^ recognize that metacommunity theory must go beyond the historically predominant thinking of considering environmental determinism as the main scenario of macrophyte community assembly. For example, Brown *et al*.^2^ emphasized the degree to which dispersal limitation interferes with environmental filtering by hindering species’ tracking of local environmental conditions. By using effective (genetic) dispersal estimates, our results are broadly in line with recent findings from metacommunity theory^20,21^ (confirming our third hypothesis, **H3**) and re-emphasize the need to go beyond the historically predominant paradigm of understanding environmental heterogeneity as the main force driving macrophyte gene flow patterns.

Studies of single species are undoubtedly valuable for population genetics, often providing greater power and resolution for examining patterns of biological variation than coarse proxies of dispersal from spatial eigenfunction analyses^14^. However, since most metacommunities comprise dozens to hundreds of species, adding additional species to the analysis would provide a big step further for examining how ecological and landscape variation shapes the distribution of genetic diversity in nature^10,14^. For the moment, our study is an important step towards integrating population genetics into the full inference space of metacommunity ecology. Different types of approaches for molecular-based studies, including large multi-species population genetic data and DNA barcoding of entire assemblages^10^, will play a major part in the next big steps, providing an exciting frontier for metacommunity ecology that may open up many advances of scientific inquiry.

In conclusion, we highlighted the influence of spatial processes on patterns of genetic differentiation in *M. alterniflorum* under a relatively wide environmental gradient in Mediterranean ponds. Despite the relatively short distances between the study ponds (mean pairwise geographic distance ~ 15 km), plant populations clustered into genetic groups that mirrored their geographic distributions. Perhaps more importantly, we found a significant correlation between genetic variation and geographic distance at the regional scale, which essentially requires that gene flow occurs primarily between nearby populations. Accordingly, these findings emphasise that dispersal limitation at the landscape level may be an additional point of major conservation concern for aquatic macrophytes. Further studies examining the processes structuring genetic variation of multiple aquatic macrophyte species are needed to demonstrate whether the pattern provided by *M. alterniflorum* is typical or anomalous for macrophytes in this kind of highly fragmented landscapes. Hence, comparative studies, either of population genetics, metacommunity ecology or both in combination, will help us obtain a deeper understanding of whether or not spatial processes may hinder aquatic macrophyte species from tracking environmental variation.

## Methods

### Site description

We performed this study on 11 ponds located in a central, lowland area (around 900 m above the sea level) of approximately 200 km^2^ in northern Spain (Supplementary Fig. S2). The predominant land uses in the study area are arable and pasture and the climate is Mediterranean dry moderate. The majority of ponds studied are fed mostly by groundwater and rainfall and experience a strong reduction in water volume during the summer, ranging between 0.7 and 4.8 ha in aerial extent and 0.3 and 1.5 m in depth. The site selection included ponds with considerable variability in environmental conditions, including morphometry, nutrient content and mineralization (Supplementary Table S4).

### Species biology

Alternateflower watermilfoil (*M. alterniflorum*) is an anchored submerged aquatic macrophyte that is native to Europe, North America and Asia^44^. This widespread aquatic species occurs in still or slow-moving, neutral to basic water (pH = 6.0–8.8) of lakes, ponds and rivers with typically nutrient-poor and fine mineral sands sometimes mixed with muck^44,45^. *M. alterniflorum* presents diverse reproduction modes dispersing through both sexual and vegetative propagules (rhizomes and plant fragments). Similarly, this macrophyte species is typically dispersed by wind, water and waterfowl^45^. Together, gamete vectors and reproductive traits of this aquatic macrophyte species are expected to result in high gene flow and dispersal rates among nearby populations^22^.

### Environmental data

Pond area (ha) was measured on high resolution aerial images with ArcMap version 10.6 (Esri, Redlands, CA, USA). Maximum depth (m) and Secchi depth (m) were recorded in the deepest area of each waterbody using calibrated sticks and a Secchi discs (diameter = 20 cm), respectively. The ratio Secchi depth:maximum pond depth was used as a variable instead of Secchi depth since most of the ponds were shallow enough to keep the disc visible up to the bottom. Hydroperiod length (i.e. water residence time) was coded as a set of dummy variables, one for each category of the variable (permanent, temporary). Several water samples were randomly collected at different depths along a shore-centre transect using a cylindrical corer (diameter = 60 mm, length = 1 m). All samples from each pond were subsequently mixed to form a single composite water sample (volume = 5 l). Conductivity and pH were measured in the field from the composite water sample using WTW probes (Xylem, Weilheim, Germany). The integrated water samples were preserved in Pyrex glass bottles at 4 °C and then analysed in laboratory following standard methods^46^ to determine total suspended solids, nitrate, ammonium, total phosphorous, soluble reactive phosphorus, chlorophyll “*a*”, chlorides and sulphates.

### Plant sampling

A total of 142 individuals of *M. alterniflorum* were collected from 11 ponds in July 2018. Six to 20 young leaf fragments from each population were randomly sampled at 2–3 m intervals to avoid collecting ramets from a single genet. Fresh leaf samples were immediately dried in allochroic silica gel in the field and then stored frozen at −80 °C before being processed further.

### DNA extraction and PCR amplification

Total genomic DNA was extracted from fresh leaf samples using the DNeasy® Plant Mini Kit (QIAGEN, Hilden, Germany) and following the manufacturer’ protocol. From the 20 microsatellite primers designed by Wu *et al*.^47^, we selected a total of nine loci with clear polymorphic and reproducible bands (Supplementary Table S5). PCR amplifications were carried out in a volume of 25 µl containing a mix of genomic DNA (1 µl), Horse-Power™ Taq Polymerase (5 U µl^−1^, 0.25 µl; Canvax Biotech, Córdoba, Spain), SSR primers (10 µM, 2 µl; Thermo Fisher Scientific, Waltham, MA, USA), dNTPs (10 mM each, 2.5 µl), buffer and Cl_2_Mg 25 mM (2.5 µl each). PCR reactions consisted of an initial denaturation period of 5 min at 94 °C, followed by 35 cycles of 94 °C for 30 s, 52–59 °C for 30 s and 72 °C for 1 min, and a final 10 min extension at 72 °C, after which the samples were preserved at 4 °C. Genotyping was performed on an ABI 3130XL (Applied Biosystems, Foster City, CA, USA) automated DNA sequencer using an internal size standard (GeneScan^TM^ 500Liz®, Applied Biosystems) for accurate sizing. Then, GeneMapper version 4.0 (Applied Biosystems) was used for allele calling.

### Genetic diversity

Genetic diversity estimates, such as allele richness (N_a_ and N_e_), total genetic diversity (H_T_), mean genetic diversity (H_S_), and observed and expected heterozygosities (H_o_ and uH_E_), were computed with GenAlEx version 6.5^48^. Nei’s unbiased genetic distance^49^ (D_A_) and Wright’s F statistics^50^, including inbreeding coefficient (F_IS_), total inbreeding (F_IT_) and fixation index (F_ST_), were also determined with the same statistical software. Clone assignment was conducted with the criterion of treating individuals with the same multilocus genotype as a clone, and only the genotypes of the genets were kept for subsequent analyses.

### Population genetic structure

The number of genetic clusters of the 11 alternateflower watermilfoil populations was assessed by using a Bayesian clustering method implemented in the software STRUCTURE version 2.3^51^. We tested *K* (i.e., the number of clusters) in ten independent runs from 1 to 11 (burn-in period of 10,000 iterations and 10,000 Markov chain Monte Carlo, MCMC, replicates in each run), without using sampling site as a prior to assess convergence of the estimated *In* probability of the data, *In P (D)*. Runs were carried out under the admixture model with independent allele frequencies. The best-fit number of clusters was calculated based on the second order rate of change of the likelihood function, *∆K*^52^. Discriminant analysis of principal components^53^ (DAPC) was also carried out as an alternative method for determining broad-scale population structure using the dapc function from the ‘adegenet’ package version 2.1.1^54^ in R. DAPC is a multivariate approach that combines principal component analysis together with discriminant analysis to summarize genetic differentiation between groups. DAPC is free of assumptions about Hardy–Weinberg equilibrium or linkage disequilibrium and provides graphical representation of the divergence among populations. The method requires *a priori* clustering algorithms determined by *k*-means. We evaluated up to *k* = 11 groups, and Bayesian information criterion^53^ (*BIC*) was used to assess the number of clusters best fitting the data. However, the value of *BIC* kept decreasing with the increase of *k*. We therefore set an identical *k* values as the *K* of STRUCTURE for comparison^16^.

We created a minimum spanning network (MSN) to illustrate patterns of gene flow between populations using EDENetworks version 2.18^55^. The method is a divisive-hierarchical clustering-like process in which the network is scanned from a fully connected state to a critical threshold distance (i.e. percolation threshold) without *a priori* assumptions of the clustering of populations. The software plots all populations as nodes in a network graph with connections (links or edges) between nodes weighted by their pairwise Nei’s unbiased genetic distance (D_A_). The layout of the MSN was recomputed 10 times to test for possible alternative network shapes.

We applied the Monmonier’s maximum difference algorithm^56^ as implemented in the package ‘adegenet’ to identify the geographic areas associated with genetic discontinuities in the study populations. The initial connection network was built using UTM coordinates for the sites. Detection of genetic discontinuities was based on the Delauney triangulation and the resulting Voronoi tessellation. Each edge of the Voronoi polygons was associated with the value of the corresponding D_A_ between pairs of populations. The algorithm then built genetic boundaries based on maximum pairwise distances^56^. Statistical confidence of the genetic barriers detected, corresponding to an abrupt change in the patterns of genetic variation among populations, was evaluated using 1,000 bootstrap replicates that were simulated with the function writeBoot from the package ‘diveRsity’ version 1.9^57^. Analyses were also conducted separately for each amplifying microsatellite locus.

### Landscape genetic analysis

We reduced the available environmental variables to a more parsimonious set by performing principal component analysis (PCA) on independent environmental attributes (Spearman’s rank correlation r_s_ < 0.7) with the princomp function from the ‘vegan’ package version 2.4^58^. After dealing with multicollinearity, local environmental attributes included: hydroperiod length, aerial extent, depth, relative Secchi depth, pH, conductivity, total suspended solids, ammonium, total phosphorous and chlorophyll “*a*”. The environmental (Euclidean) distances between populations were calculated from the values obtained from the PCA-based model selection procedure. Both geographic (Euclidean distances between pond UTM coordinates) and environmental distance matrices were constructed using the vegdist function from the ‘vegan’ package. The correlations between geographic/environmental factors and Nei’s unbiased genetic distances were assessed by a combination of partial Mantel tests^59^ and multiple matrix regression with randomization^60^ (MMRR). Partial Mantel tests with 10,000 permutations were performed between genetic distances and one factor under the influence of the other (as covariate) using the mantel.partial function implemented in the ‘vegan’ package. Similarly, MMRR was implemented with 10,000 iterations to estimate the independent effect of geographic/environmental factors using the MMRR function script^60^. The main advantages of this method are that it produces appropriate levels of Type-I error^60^, and it uses multiple regression, assessing the independent contribution of each variable in the model.

## Supplementary information

Supplementary Information

## Data Availability

The data sets generated during and/or analysed during the current study are available from the corresponding author on reasonable request.
