# Supplementary material for: Bridging population genetics and the metacommunity perspective to unravel the biogeographic processes shaping genetic differentiation of Myriophyllum alterniflorum DC
Source: Sci Rep. 2019 Dec 2;9:18097. doi: 10.1038/s41598-019-54725-7 (PMC6889409; doi:10.1038/s41598-019-54725-7)
Supplement: Supplementary file 1 — Supplementary Information [file 41598_2019_54725_MOESM1_ESM.pdf]

Scientific Reports

SUPPLEMENTARY INFORMATION

Bridging population genetics and the metacommunity perspective to unravel the biogeographical processes shaping genetic differentiation of *Myriophyllum alterniflorum* DC.

Jorge García-Girón[1*], Pedro García[2], Margarita Fernández-Aláez[1], Eloy Bécares[1], Camino Fernández-Aláez[1]

[1]Group for Limnology and Environmental Biotechnology, Area of Ecology, Universidad de León, Campus de Vegazana, León, Spain.

[2]Department of Molecular Biology, Universidad de León, Campus de Vegazana, León, Spain.

* Correspondence and requests for materials should be addressed to **J.G.G.** (jogarg@unileon.es)

Jorge García-Girón: jogarg@unileon.es **ORCID ID:** 0000-0003- 0512-3088

Pedro García: pgarg@unileon.es **ORCID ID:** 0000-0002-3800-7326

Margarita Fernández-Aláez: mfera@unileon.es **ORCID ID:** 0000-0003- 3847-6613

Eloy Bécares: ebecm@unileon.es **ORCID ID:** 0000-0002-7123-3579

Camino Fernández-Aláez: mcfera@unileon.es **ORCID ID:** 0000-0001- 9385-1354

Telephone number: 34987291569

Fax number: 34987291563

Table S1 Pairwise Nei´s unbiased genetic distance (D_A) between 11 *Myriophyllum alterniflorum* populations.

	AMO	LIN	SE	MAN	CAR	MAY	DIE	CAN	RAQ	SEN
LIN	0.058									
SE	0.262	0.130								
MAN	0.620	0.372	0.396							
CAR	0.368	0.304	0.101	0.139						
MAY	0.325	0.219	0.190	0.163	0.041					
DIE	0.399	0.185	0.221	0.048	0.035	0.060				
CAN	0.457	0.334	0.229	0.113	0.037	0.026	0.059			
RAQ	0.485	0.431	0.251	0.142	0.017	0.046	0.082	0.010		
SEN	0.560	0.508	0.307	0.109	0.091	0.107	0.124	0.042	0.019	
ERA	0.539	0.443	0.221	0.133	0.054	0.075	0.101	0.016	0.010	0.011

Table S2 Number of genets for each population assigned to each of the two genetic clusters based on the DAPC analysis.

Populations	Number of genets	Number of genets associated to the southern cluster	Number of genets assigned to the northern cluster
AMO	13	13	0
LIN	9	9	0
SE	8	7	1
MAN	16	0	16
CAR	6	2	4
MAY	14	5	9
DIE	8	2	6
CAN	20	2	18
RAQ	11	0	11
SEN	20	1	19
ERA	17	1	16

Table S3 Contribution (%) of independent (Spearman rank-order correlation $r_s < 0.7$) environmental attributes to the principal component analysis. Only the first two synthetic axes (PCA1 and PCA2; in bold) were used to compute the environmental (Euclidean) distances between populations. Prop. = the proportion of the variance (%) explained by each principal component.

	**PCA1**<br>**Prop. = 67.14**	**PCA2**<br>**Prop. = 31.10**	PCA3<br>Prop. = 1.22	PCA4<br>Prop. = 0.41	PCA5<br>Prop. = 0.11
Hydroperiod length	16.31	3.74	9.29	15.93	0.14
Area	0.27	10.49	24.51	13.04	4.42
Max. depth	9.51	2.74	15.81	8.33	27.95
Relative Secchi depth	2.75	15.11	9.37	18.85	10.07
pH	5.83	30.14	.84	0.79	0.40
Conductivity	10.17	15.95	4.65	3.39	8.94
Total suspended solids	18.54	0.037	7.87	2.15	31.67
Ammonium	17.20	3.25	0.22	21.30	10.61
Total phosphorous	14.57	4.95	11.95	0.02	0.95
Chlorophyll "*a*"	4.84	13.59	13.38	16.17	4.85

Table S4 Mean and range of environmental attributes in the study ponds.

Variable	Mean	Range
Area (Ha)	1.91	0.69-4.79
Max. Depth (cm)	52.5	1.5-140
Relative Secchi depth	0.78	0.31-1
pH	7.12	4.94-8.67
Conductivity (µS cm^{-1})	396	90-770
Total suspended solids (mg l^{-1})	9.08	1.14-25.96
Nitrate (mg l^{-1})	0.16	0.00-1.20
Ammonium (µg l^{-1})	53.4	23.8-114.5
Total phosphorous (mg l^{-1})	160.7	25.9-796.8
Soluble reactive phosphorous (µg l^{-1})	39.9	3.6-207.3
Chlorophyll "*a*" (mg l^{-1})	12.5	1.7-43.2
Chlorides (mg l^{-1})	35.6	2.9-110
Sulfates (mg l^{-1})	13.8	0.9-57.7

Table S5 Characteristics of the nine microsatellite loci used in this study. Note: T_a = annealing temperature. All forward primers were labelled with fluorescent dyes.

Locus	Primer sequence	Repeat motif	Range (bp)	T_a (°C)	Fluorescent dye	GenBank accession no.
Myrsp1	F: GTCAAAGCAGCCACTCGG<br>R: GGCAACAATGCAGCTAACC	$(TCA)_3(TCAGCA)_2(GCA)_3$	159-186	59	6-FAM	JX000192
Myrsp4	F: ACTGGCTAATGATATGCTGA<br>R: TCTTTCCACGCCTCTTC	$(TC)_{17}(AC)_9$	250-290	54	VIC	JX000195
Myrsp6	F: TAACAAACCGTACATTACAAGC<br>R: TTTCTCTGGGAGCCATAAC	$(TC)_{17}$	136-152	59	6-FAM	JX000197
Myrsp9	F: TCCCCATCTGGTTCGTAT<br>R: GGAAGGTAGCGGAGTGC	$(ATC)_5(TTCATC)_2(TTC)_2$	213-237	58	6-FAM	JX000200
Myrsp12	F: CGCTTCACAAGTATTCTG<br>R: TTCATGGTAGCCGTCA	$(TC)_{18}(AC)_{10}$	362-394	52	PET	JX000203
Myrsp13	F: GCTTCCATTGCGAAACTT<br>R: CCCAAACACCACCTCATT	$(GCA)_4(TCA)_{4(}GCA)_3$	432-468	55	PET	JX000204
Myrsp14	F: TTCCCATCCTTCTCCTG<br>R: CCAAGTAAGTGTCCCAAAC	$(TA)_2(TG)_8(TA)_8(GA)_4$	286-308	58	6-FAM	JX000205
Myrsp15	F: TCTTTCCACGCCTCTTC<br>R: ACTGGCTAATGATATGCTGA	$(TG)_7(AG)_9$	260-290	56	VIC	JX000206
Myrsp16	F: GGCTGCCCTATGCTAA<br>R: ATCCCACTGAAGTCAAACT	$(TG)_2(CA)_8(TA)_6(GA)_6$	320-364	54	PET	JX000207

Figure S1 Modelling of the number of genetic clusters in *Myriophyllum alterniflorum* using STRUCTURE. The mean (±SD) log probabilities of the data (*In P (D)*; right axis, open dots and error bars) and the magnitude of *ΔK* (left axis, BALCK line), calculated according to Evanno *et al.*[1], plotted against the number of modeled genepools (*K*).

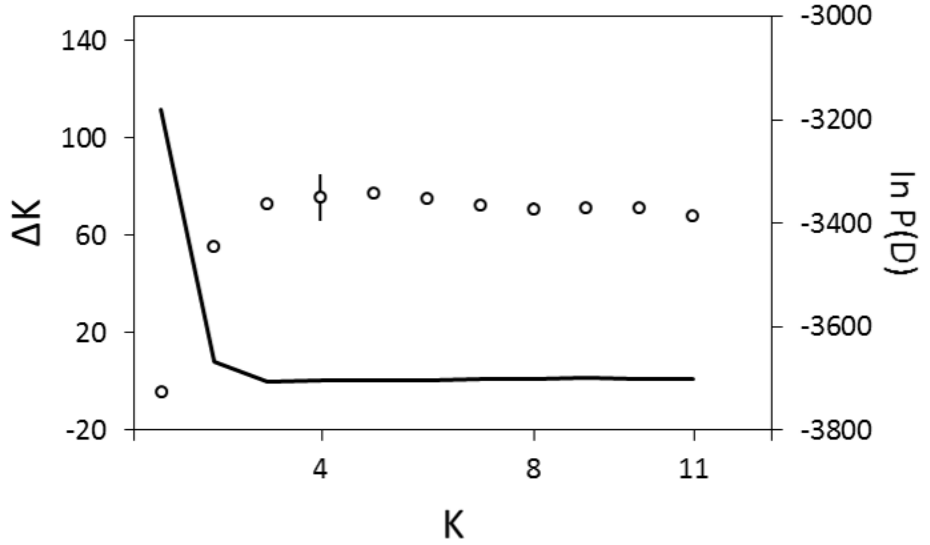

Figure S2 Location map of study sites.

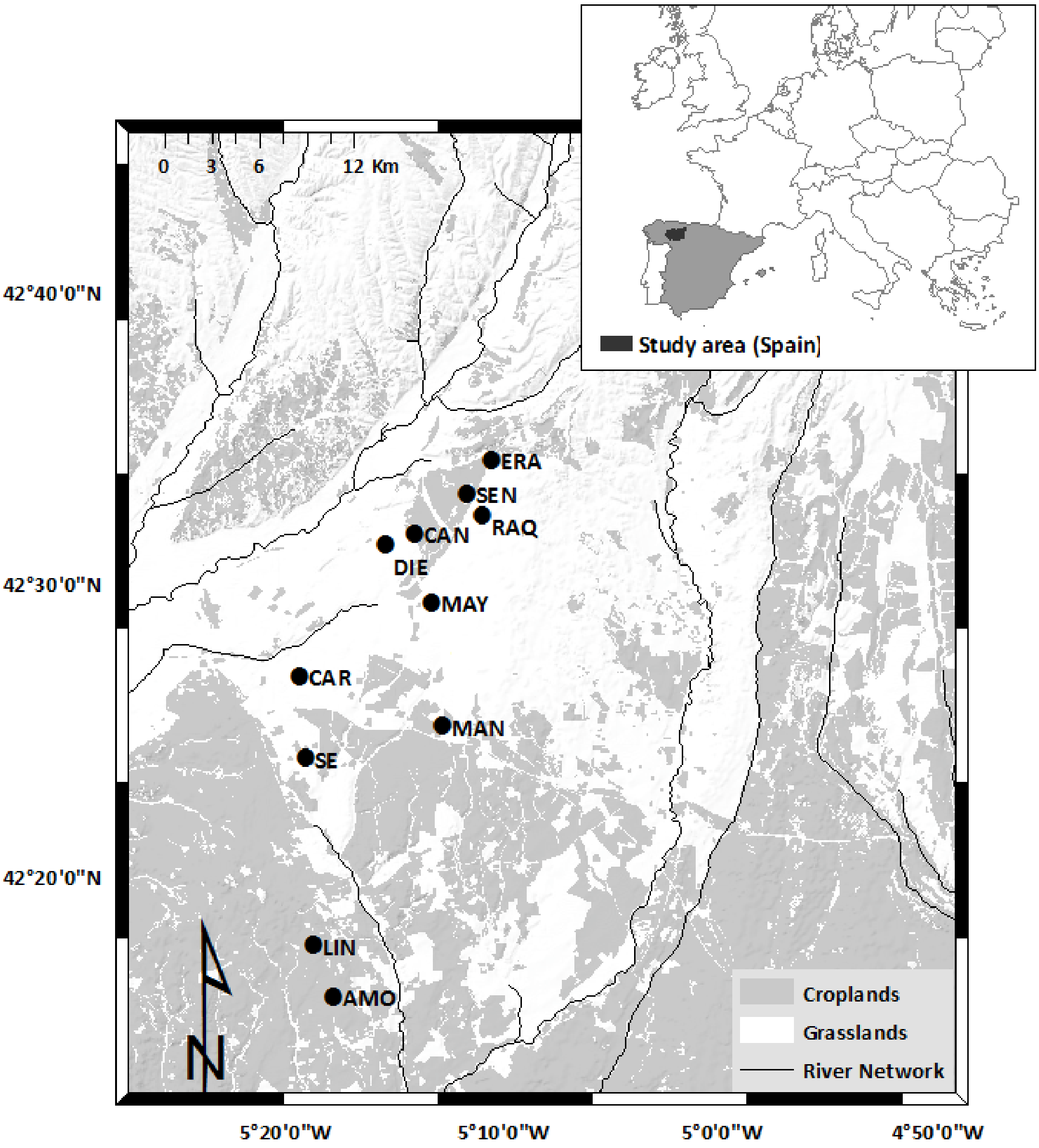

References

1. Evanno, G., Regnaut, S. & Goudet, J. Detecting the number of clusters of individuals using the software STRUCTURE: a simulation study. *Mol. Ecol.* **14**, 26-2620 (2005).
